# Intervention Design Elements Are Associated with Frontline Health Workers’ Performance to Deliver Infant and Young Child Nutrition Services in Bangladesh and Vietnam

**DOI:** 10.1093/cdn/nzz070

**Published:** 2019-07-10

**Authors:** Phuong Hong Nguyen, Sunny S Kim, Lan Mai Tran, Purnima Menon, Edward A Frongillo

**Affiliations:** 1 Poverty, Health and Nutrition Division, International Food Policy Research Institute (IFPRI), Washington, DC, USA; 2 Department of Health Promotion, Education, and Behavior, University of South Carolina, Columbia, SC, USA

**Keywords:** frontline workers, intervention design element, knowledge, motivation, performance, Bangladesh, Vietnam

## Abstract

**Background:**

Frontline health workers (FLWs) are needed for delivering interventions at scale to reduce maternal and child undernutrition, but low- and middle-income countries often face inadequate FLW performance.

**Objectives:**

We examined whether and how intervention design elements such as training, supervision, and mass media improved FLW performance in delivering nutrition services.

**Methods:**

Survey data were collected in 2010 and 2014 as part of impact evaluations of Alive & Thrive (A&T) interventions to improve infant and young child feeding (IYCF) practices in Bangladesh and Vietnam. FLWs in A&T intensive (A&T-I) areas received specialized IYCF training, job aids, and regular supportive supervision. Those in non-intensive (A&T-NI) areas received standard government training and supervision. There was mass media exposure in both areas. Multiple regression was used to test differences in exposure to intervention design elements and performance outcomes between the 2 program areas. Path analyses were conducted to examine the paths from exposure to performance outcomes measured at FLW and end-user levels.

**Results:**

Compared to FLWs in A&T-NI areas, those in A&T-I areas had higher scores in training (by 1.3–3.6 of 10 points), supportive supervision (0.3–3.5 points), and mass media exposure (0.3–3.5 points). These intervention design elements were significantly associated with FLW knowledge and motivation, which in turn improved service delivery. FLW-level performance outcomes contributed to improving end-user-level outcomes such as higher service received (β = 0.12–1.04 in Bangladesh and 0.11–0.96 in Vietnam) and maternal knowledge (β = 0.12–0.17 in Bangladesh and 0.04–0.21 in Vietnam).

**Conclusions:**

Training, supervision, and mass media exposure can be implemented at large scale and contribute to improved FLW service delivery by enhancing knowledge and motivation, which in turn positively influence mother's service utilization and IYCF knowledge. Training, supervision, and mass media to enhance service provision should be considered when designing interventions. This trial was registered at clinicaltrials.gov as NCT01678716 (Bangladesh) and NCT01676623 (Vietnam).

## Introduction

Millions of mothers and children die unnecessarily each year due to undernutrition (1, 2), mostly in low- and middle-income countries (LMICs). Reducing maternal and child undernutrition requires implementation of effective health and nutrition interventions at scale (3). To deliver these interventions, frontline workers (FLWs), paid or volunteer, at the local health facility or community levels, are the first and often the only point of contact with the health care system for

millions of people (4). FLWs are considered the backbone of the health system and serve as the conduits of information, resources, and primary care services and counseling. Systematic reviews have shown, however, that LMICs face inadequate performance of FLWs, which in turn harms service quality (5). Thus, understanding the factors influencing FLW performance is needed to improve service provision and to strengthen health systems.

There is increasing evidence of multiple factors that influence the performance of FLWs in LMICs, including contextual factors that are not easily adjustable (6) and intervention design factors that are modifiable (7). Contextual factors (including the community, political and economic contexts, environment, and health systems) were found to interact with each other to influence FLW performance as well as program performance (6, 8, 9). FLW characteristics such as age, gender, social status, and work experience may also be associated with performance (4, 10), or they may modify the effects of interventions on FLW performance, although evidence on these factors is inconclusive (6). Sociocultural factors (e.g., women's education, gender roles, and community values and norms) are associated with women's access to and uptake of services and thereby influence FLW performance, especially in maternal health programs (6).

Although political and community contexts and health systems are important determinants of FLW performance, intervention design elements have gained more attention because they can be directly shaped and adjusted. A review of 140 studies on FLW programs reported that a variety of intervention design elements (e.g., training, supervision, and incentives) can strengthen FLW performance (7), although the influence of other factors related to the use of guidelines and protocols, performance appraisal, and some aspects of monitoring and evaluation is not clear. Both pre-service and in-service training can help provide and maintain a good level of knowledge and skills, which affects the ability of FLWs to adhere to service standards, procedures, and practices (11–13). The learning process involving training content, learning methods, and feedback from others influenced not only individual cognition but also affective attributes (e.g., self-efficacy, problem-solving, and communication skills) (14). Regular continuous training had a positive effect on FLW performance, yet there was no evidence regarding the length of training (7). Although supervision has a number of indirect effects on job satisfaction and motivation, retention, and skills development, its role in improving the quality of care and health outcomes is uncertain (15). In addition, although financial incentives have been shown to increase motivation and performance of FLWs, nonfinancial incentives (e.g., community trust, respect, and recognition) also played critical roles in enhancing motivation, self-assessed performance, and adherence to guidelines (7). Motivation can critically influence FLW performance, either directly or through mediating effects on service quality, provision, and efficiency (5, 16), by limiting the willingness to exert and maintain an effort to do work.

FLWs have demonstrated effective delivery of services related to immunization promotion, improved outcomes for selected infectious diseases, and reduced childhood morbidity and mortality (17). Evidence on the performance of FLWs on promoting IYCF practices is inconsistent, however, due to substantial heterogeneity in different settings and cultural norms or traditions (17). Alive & Thrive (A&T) is an initiative that mobilized existing FLWs from different types of health systems—a nongovernmental organization (NGO) in Bangladesh and government in Vietnam—to deliver IYCF counseling and other basic health services at health facilities or through home visits, food demonstrations, and health and nutrition forums at the community level. During a 4-y period (2010–2014), program interventions led to large significant impacts on IYCF practices in both Bangladesh (18, 19) and Vietnam (20). This article examines 2 research questions: Did the A&T interventions improve FLW performance in delivering nutrition services in Bangladesh and Vietnam? and How did the A&T interventions improve FLW performance?

## Methods

### Study context and intervention description

This study is part of the overall impact evaluation of A&T's intervention programs to improve IYCF practices in Bangladesh and Vietnam (21). Detailed descriptions of the interventions, evaluation design, data collection, and main results have been reported elsewhere (19, 20, 22). Briefly, A&T is an initiative that aims to save lives, prevent illness, and contribute to healthy growth and development through improving IYCF practices. From 2009 to 2014, A&T operated in Bangladesh and Vietnam, reaching millions of children aged <2 y through large-scale social and behavior change communication interventions. A core component of the program was to mobilize existing FLWs (health workers and community volunteers) to deliver IYCF counseling and other basic health services at health facilities or through home visits, food demonstrations, and health and nutrition forums at the community level.

In Bangladesh, A&T in collaboration with BRAC, a large NGO, facilitated the training of >75,000 health workers [called *Shasthya Kormi* (SK)] and volunteers [called *Shasthya Sebika* (SS)] throughout the country. Additional IYCF paid promoters, called *Pushti Kormi* (PK), were recruited and trained to provide more skilled support for breastfeeding and complementary feeding (23). Each SS, accompanied by a PK, conducted home visits as their daily tasks to deliver multiple age-targeted IYCF-focused counseling to pregnant women and mothers of children up to age 2 y, coached mothers as they tried out the practices, and engaged other family members to support the behaviors. Each SS visited ∼15 households in her neighborhood per day, 5 d/wk, to ensure all mothers with children aged <24 mo were visited once a month. One PK accompanied 1 SS each day during the SS household visits to ensure the following schedule of contacts with mothers during the first 2 y: monthly from 0 to 8 mo, every other month from 9 to 12 mo, and 1 more visit each between 15 and 18 mo and 23 and 24 mo. During the home visits, the PK also engaged other family members in discussion to get their support for the recommended IYCF practices. The SKs had a much larger area (2500–3000 households) to cover compared with the SSs, who worked only in their own neighborhood (250–300 households); therefore, the SKs visited each household in their catchment area only twice a year. The SKs spent most of their time conducting antenatal and postnatal care sessions at home, and they used the time they were in contact with pregnant women and mothers to promote and support good IYCF practices. The SKs also invited pregnant women, mothers, and other family members to attend monthly food demonstrations or health forums on a variety of topics. Health forums were normally held in a courtyard or on the verandah of a home. The SKs were expected to conduct 4 or 5 forums per week, each lasting ∼30 min.

In Vietnam, A&T with Save the Children, worked with the government to establish a total of 781 social franchises within government health facilities in 15 of 63 provinces at the province, district, and commune levels to improve the quality of IYCF counseling. Nearly 2000 health staff (HS) were trained to work as counselors to deliver IYCF counseling services, and 7000 village health workers (VHWs) distributed invitation cards to target beneficiaries to create demand. The HS was expected to deliver 5 major components of the IYCF service package: *1*) exclusive breastfeeding promotion at the third trimester of pregnancy with 3 contacts, *2*) exclusive breastfeeding support at the time of delivery with 1 contact, *3*) exclusive breastfeeding management when the child was 0–6 mo with 4 contacts, *4*) complementary feeding education when the child was 5–6 mo with 1 contact, and *5*) complementary feeding management when the child was 6–24 mo with 6 contacts. Thus, each mother–child pair was expected to receive a maximum of 15 contacts during a 27-mo period.

In addition to training, FLWs in both countries were exposed to mass media, which consisted of nationally broadcasted TV spots that targeted mothers, family members, health workers, and local doctors with messages on various aspects of IYCF. In both countries, the programs also focused on monitoring and supportive supervision for FLWs and provided job aids and training materials; in Bangladesh, the volunteers received performance-based cash incentives (24, 25).

### Study design and participants

This study used a cluster-randomized impact evaluation design in which 20 rural subdistricts in Bangladesh and 40 communes in Vietnam were randomly assigned to either *1*) A&T–intensive areas (A&T-I), which received intensified counseling on IYCF, mass media, and community mobilization; or *2*) A&T–non-intensive areas (A&T-NI), which received standard nutrition counseling and less intensive mass media. In A&T-I areas, FLWs received full and refresher training specifically on IYCF topics, job aids, and regular supportive supervision (24, 26–28). In A&T-NI areas, FLWs received standard government training with irregular supervision. FLWs in both areas were exposed to mass media. Detailed differences between A&T-I and A&T-NI areas are presented in **Supplemental Tables 1** and **2**.

Survey data from FLWs and households were collected through 2 cross-sectional surveys in 2010 (baseline) and 2014 (endline) in the same communities (between June and August at both time periods). All baseline characteristics and performance of FLWs were balanced between the 2 program areas. This article focuses on FLWs directly involved in service delivery, and thus it uses the endline survey data only. In Bangladesh, within each of the 20 subdistricts, 5 unions and 2 villages within each union were randomly selected to yield a total of 200 villages. All FLWs working in these villages were included in the survey; thus, data were collected for a total of 107 health workers (SK) and 302 health volunteers (SS). In Vietnam, 3 HS who were primarily responsible for IYCF services in the commune health center and all VHWs in the study areas were selected, yielding a total sample of 120 HS and 327 VHWs. The household sample was selected based on systematic random sampling from household census list and included 1000 mothers with children aged <2 y in each program group in each country. Data were collected via face-to-face interviews using structured questionnaires. Ethical approval was obtained from the Institutional Review Board of the International Food Policy Research Institute in the United States, the Medical Research Council in Bangladesh, and the Union of Science and Technology Association in Vietnam.

### Conceptual framework

The selection of determinants was guided by a conceptual framework of factors influencing FLW performance developed from systematic reviews of various studies in LMICs (6–8). FLW performance is a function of both intervention design elements and individual characteristics (FLW and end users). In our study, intervention design elements were measured as IYCF training received, supportive supervision received, incentive received (for Bangladesh), and exposure to mass media (**Supplemental Table 3)**. These interventions were expected to improve FLW knowledge and motivation, which in turn would improve their performance in terms of quantity and quality of services provided. FLW-level performance outcomes also contribute to end-user-level outcomes; thus, FLW performance was assessed at both the FLW level (as knowledge, motivation, and service delivery) (**Supplemental Tables 4A–4C)** and end-user level (as mothers’ knowledge and service utilization) (**Supplemental Tables 5A** and **5B**).

### Measures

#### Performance

Performance measures at the FLW level were FLW knowledge, motivation, and service delivery. FLW knowledge about IYCF was assessed based on their response to 13 questions related to breastfeeding (e.g., early initiation, colostrum, and exclusive breastfeeding) and 10 questions related to complementary feeding (e.g., timely introduction, meal frequency, and dietary diversity). All the knowledge items corresponded to the expected knowledge changes after receiving A&T's training and WHO guidelines of optimal IYCF. These measures were pretested in the field and then revised and adapted to be appropriate with the local contexts. Each question was given a score of 1 or 0 depending on a correct or incorrect response, respectively. An overall score of correct knowledge was generated separately for each country and for each type of FLW.

FLW motivation was measured by a set of 24 items that reflected 7 dimensions: confidence and enjoyment of work, personal recognition or appreciation, career training and continuing education, satisfaction with salary, support from colleges and supervisors, work pressure, and thoughts of leaving and negative attitude. These items were validated in a study in rural Haiti (29) and were piloted and adapted for relevance for Bangladesh and Vietnam. FLWs were asked to indicate their degree of agreement with each statement or, where relevant, for an estimate of how often they had experienced the sentiment echoed in the statement, scored on a 5-point Likert scale. A score of 5 represented the response “strongly agree” for positively worded statements, whereas negative statements were coded in the opposite direction so that a score of 5 represented “strongly disagree.” Cronbach’s α coefficients for different FLW types in this study ranged from 0.69 to 0.83. An overall summative score of motivation was created for each type of FLW in each country.

Service delivery was measured by both quantity and quality of service provision. In Bangladesh, FLWs were asked to report the numbers of households they visited and the number of health and nutrition forums and food demonstrations conducted each month. In Vietnam, FLWs reported the number of clients to whom they provided IYCF counseling; FLWs were able to refer to their log books during the interview to help minimize social desirability bias. Among VHWs, performance was assessed by the total number of invitation cards delivered to different targets: pregnant women and mothers with children <6 mo or 6–24 mo. For service quality, in both countries, FLWs reported the number of topics delivered during counseling sessions with children in different age groups or during health forums. These topics were based on the guideline for IYCF counseling from implementation manuals for Bangladesh (30) and Vietnam (31). The total performance score was created for each FLW type.

Performance measures at the end-user level included maternal knowledge and service utilization, which were constructed from household data. Maternal knowledge on IYCF was created in a similar way and using the same sets of items as for FLW knowledge. Service utilization included both exposure to services and quality of counseling service. In Bangladesh, mothers were asked how many visits they had received during the third trimester of pregnancy and during childhood; they were also asked about the number of visits during the past 6 mo and past 30 d. In Vietnam, mothers were asked for the number of facility visits during pregnancy and different periods of childhood (<6 mo, 6–12 mo, and 12–24 mo); they were also asked about the number of visits during the past 6 mo and when was the most recent visit. For the quality of services, mothers were asked about the topics of counseling received during the last visit in both countries. These topics needed to be appropriate for the child's age and were based on the guideline for IYCF counseling from the implementation manual for Bangladesh (30) and Vietnam (31). Each message received was given a score of 1, and the sum was used as the quality of counseling service score.

#### Intervention design elements

Intervention design elements were measured as IYCF training received, perception about supervision, and exposure to mass media. The training score was constructed based on FLWs’ participation in full and refresher training and the number of IYCF topics received from full training, monthly refresher, and the last refresher training. Different training elements were administered to FLWs based on their roles and responsibilities. Total training score was created by summing scores from these items. FLWs’ perceptions about supportive supervision were measured by a set of 17 items related to feedback, exchange and support to manage stress, and workload (Supplemental Table 1). FLWs were asked to indicate their degree of agreement with each statement using responses on a 5-point Likert scale. Factor analysis was conducted to confirm the structure of the supervision items. Cronbach’s α coefficients for different types of FLWs ranged from 0.74 to 0.88, indicating adequate internal consistency of items in the scales. A summative scale was constructed for the supervision score. Performance-based incentives (for SSs in Bangladesh) was measured as whether SSs received money incentives and for what activities SSs received incentives (e.g., ensuring high coverage, carrying out age-appropriate counseling at home visits, and maternal reports of practicing the recommended behaviors). In Vietnam, given that VHWs in Vietnam received only certificates of recognition (which most of them did), we did not include performance-based incentive as part of the Vietnam program. Mass media exposure was assessed by reported exposure to various IYCF TV spots and the number of messages recalled. We created a score based on the number of messages that FLWs recalled from the TV spots.

#### Individual characteristics

FLW characteristics included age, education, and job duration. Because FLWs’ educational levels differed between the 2 countries, we used a common categorization for education (never attended school, primary school, secondary school, high school, college, and university), but we used different reference groups as suitable for each type of FLW and each country. Mothers’ (i.e., end users’) characteristics included age, education, socioeconomic status (SES), decision-making power, and time pressure. The SES index was constructed using a principal component extracted from multiple variables, including housing materials and ownership of house, land, and various assets and livestock (32, 33). Decision-making power was measured by mothers solely or jointly making the decisions in major purchases, cooking, mobility, health care visit, and care of children including child feeding. Time pressure was measured by asking women if they believed they had enough time to take care of the child, finish office or field work, and finish daily household work, and how often they felt pressure with these chores. A score was assigned for each decision-making activity or each task women mentioned as having time pressure, and a sum of scores was created.

### Data analysis

All the scales for intervention design elements and performance outcomes were standardized to a range from 0 to 10 for comparability. All analyses were conducted separately for each type of FLW in each country. Descriptive analysis was used to examine the characteristics of the study samples. Differences in intervention design elements and performance outcomes between the 2 program groups were tested using linear regression models, adjusting for geographic clustering (34). Multiple linear regression analyses were conducted to examine the association between individual characteristics and FLW performance, using data from A&T-I areas only. To examine the paths from exposure to intervention design elements to performance outcomes measured at the FLW and end-user levels, we aggregated the FLW data with the household data to the village level. Path analyses were used to examine the paths of exposure to different A&T intervention elements on FLW performance as well as the linkages between FLW and end-user-level outcomes, adjusting for individual characteristics of FLWs and mothers and geographical clustering. Statistical analysis was performed using Stata version 15 software. Statistical significance was defined as *P* < 0.10 for the path analyses and *P* < 0.05 for all other analyses.

## Results

### Sample characteristics

FLWs were younger in Bangladesh compared with Vietnam (average age: 31 y compared with 42 y for health workers and 41 y compared with 45 y for volunteers, respectively) (**Table 1**). Average time working in the health system was longer for FLWs in Vietnam. Whereas all FLWs in Bangladesh were women, one-fifth of FLWs in Vietnam were men. The level of education differed depending on FLW type and country.

**TABLE 1:** Sample characteristics in Bangladesh and Vietnam^1^

Characteristics	Bangladesh		Vietnam	
End users	Mothers (*n *= 2001)		Mothers (*n *= 2012)	
Age, y	25.01 ± 5.35		28.20 ± 5.31	
Education				
Never attended school	13.29		—	
Primary school	30.68		8.00	
Secondary school	43.18		42.10	
High school	12.84		28.78	
College/university	—		21.12	
Socioeconomic status^2^	−0.002 ± 0.89		−0.002 ± 0.91	
Decision-making power (range: 0–10)	4.70 ± 3.16		9.40 ± 1.01	
Time pressure (range: 0–10)	3.20 ± 2.07		3.66 ± 1.76	
Frontline workers	SK (*n *= 107)	SS (*n *= 302)	HS (*n *= 120)	VHW (*n *= 327)
Age, y	30.41 ± 5.60	40.92 ± 11.71	41.56 ± 7.47	44.52 ± 10.16
Women, *n*	100	100	77.50	80.43
Work duration, y	4.87 ± 3.40	6.95 ± 5.69	12.79 ± 7.79	9.28 ± 6.91
<2	23.85	17.22	10.00	9.79
2 to <5	31.19	32.78	8.33	16.21
5 to <10	30.28	18.87	18.33	26.30
10 to <15	14.68	31.13	20.83	33.03
≥15	—	—	42.50	14.68
Education^3^				
Never attended school	—	23.51	—	—
Primary school	7.34	46.36	—	—
Secondary school	75.23	30.13	—	47.09
High school	17.43	—	—	36.39
College	—	—	70.83	16.51
University	—	—	29.17	—
Type of health workers	BRAC SK	BRAC SS	Commune health staff	Village health worker
Paid/volunteer	Paid	Volunteer (with performance-based incentives)	Paid	Volunteer
Locations of service provision	At home and community	At home and community	At facility	At home and community
Primary task	Provide antenatal and postnatal care, and health forums on a range of topics	Provide counseling on a broad range of health topics, treat basic ailments, and sell health commodities	Provide antenatal and postnatal care, other medical services, and counseling about IYCF	Conduct home visits to deliver government program through family planning services and health education; create awareness for IYCF services

1 Values are means ± SDs or percentages. HS, health staff; IYCF, infant and young child feeding; SK, Shasthya Kormi; SS, Shasthya Sebika; VHW, village health worker.

2 The socioeconomic status index was constructed using a principal component extracted from multiple variables, including housing materials and ownership of house, land, and various assets and livestock.

3 Because women's educational levels varied for each type of FLW across the 3 countries, we applied different cutoffs suitable for each type of FLW. College refers to 2 y of education after high school; university refers to 4–6 y of education after high school.

Mothers in Bangladesh were slightly younger (25 y compared with 28 y), had lower decision-making power scores (4.7 compared with 9.4), and had less time pressure (3.2 compared with 3.7) compared with mothers in Vietnam. Whereas 13% of mothers in Bangladesh had no education and 31% completed primary school only, more than 90% of mothers in Vietnam had secondary school education or higher. The characteristics of FLWs and mothers were balanced between A&T-I and A&T-NI areas (results not shown).

### Intervention design elements

**FIGURE 1:**
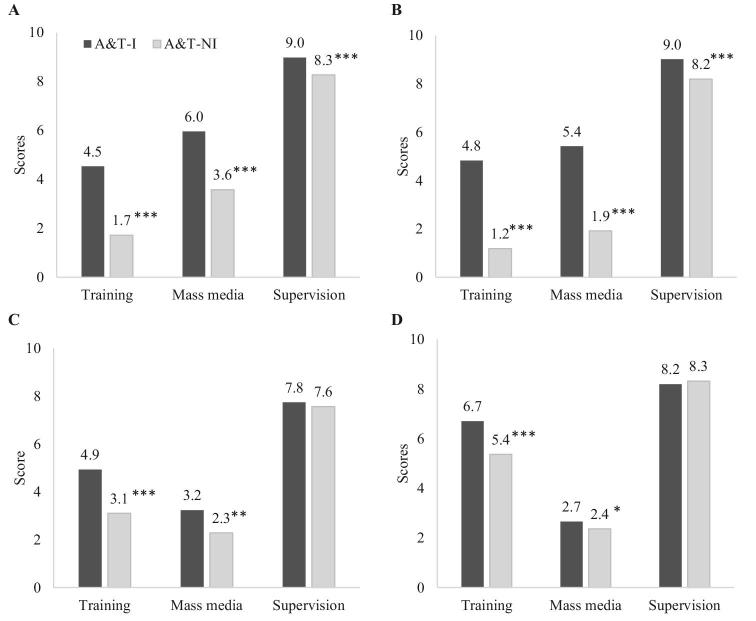
Exposure to intervention design elements among frontline health workers, by program group, in Bangladesh and Vietnam. Bangladesh SK (A), Bangladesh SS (B), Vietnam HS (C), and Vietnam VHW (D). All intervention exposures are presented as scores with a range of 0–10 (see Supplemental Table 1 for details). *, **, ***Significant difference between intensive and non-intensive areas, accounting for geographic clustering: *P < 0.05, **P < 0.01, ***P < 0.001. A&T-I, Alive & Thrive–intensive; A&T-NI, Alive & Thrive–non-intensive; HS, health staff; SK, Shasthya Kormi; SS, Shasthya Sebika; VHW, village health worker.

The percentages of FLWs in both countries who received full training was high in A&T-I areas (>80%) and much higher compared with A&T-NI areas (Supplemental Table 3). More FLWs in A&T-I areas in Bangladesh attended monthly refresher trainings compared with their counterparts in A&T-NI areas. Training content was also more comprehensive in A&T-I than in A&T-NI areas in both countries, with more topics on breastfeeding positioning and attachment, feeding techniques, feeding sick children, and problem-solving for difficulties in IYCF. VHWs in A&T-I areas received more training on food demonstrations compared with those in A&T-NI areas in Vietnam. Overall scores for IYCF training were significantly higher in the A&T-I group compared with the A&T-NI group for all types of FLWs in both Bangladesh and Vietnam, with differences ranging from 1.3 to 3.6 points (**Figure 1**).

Perceptions about supportive supervision were generally high (scores ranged from 8 to 9 out of 10), with the A&T-I group in Bangladesh scoring 0.7–0.8 points higher and no difference between groups in Vietnam. FLWs’ exposure to the TV spots was higher in A&T-I areas compared with A&T-NI areas for SSs in Bangladesh (87% compared with 58%) but did not differ between groups for SKs (85–88%) (Supplemental Table 3). Nearly all SSs in Bangladesh received monthly incentives for several activities they conducted, measured by the number of mothers showed appropriate feeding practices. Exposure to TV spots was also higher for A&T areas in Vietnam. The scores for message recall were significantly higher in the A&T-I group compared with the A&T-NI group for all FLW types (Figure 1).

### FLW performance

#### Performance measured at the FLW level

**FIGURE 2:**
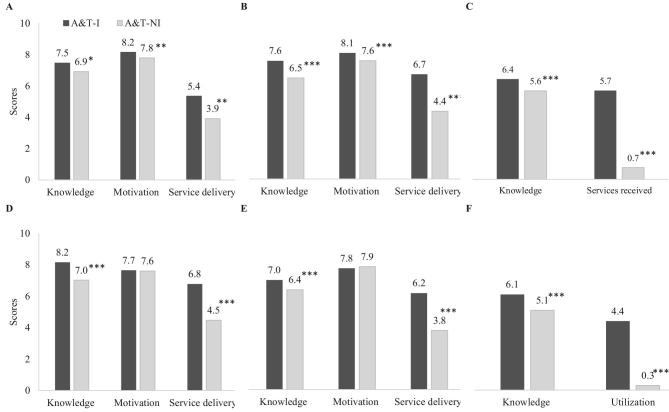
Performance measured at frontline worker and end-user levels, by program group, in Bangladesh and Vietnam. Bangladesh SK (A), Bangladesh SS (B), Bangladesh mothers (C), Vietnam HS (D), Vietnam VHW (E), and Vietnam mothers (F). *, **, ***Significant difference between intensive and non-intensive areas, accounting for geographic clustering: *P < 0.05, **P < 0.01, ***P < 0.001. A&T-I, Alive & Thrive–intensive; A&T-NI, Alive & Thrive–non-intensive; HS, health staff; SK, Shasthya Kormi; SS, Shasthya Sebika; VHW, village health worker.

FLW knowledge about IYCF was similar in Bangladesh and Vietnam and higher in A&T-I than in A&T-NI areas (**Figure 2**). The overall IYCF knowledge scores were 7.0–8.2 in A&T-I areas compared with 6.4–7 in A&T-NI areas (∼0.5–1.2 points higher on a 10-point scale). Several knowledge items were also significantly greater among FLWs in A&T-I areas, including knowing the reasons why baby should be breastfed, breastfeeding more often, not feeding baby <6 mo water even in hot weather, exclusively breastfeeding until the age of 6 mo, leaving breastmilk for baby when mother is away, and being committed to breastfeed until the age of 24 mo and beyond (Supplemental Table 4a).

FLWs uniformly noted high motivation in their work, reflected by high mean scores in several domains (Supplemental Table 4b) and in total scores (Figure 2). In Bangladesh, FLWs in A&T-I areas had higher motivation scores compared with those in A&T-NI areas (8.1 compared with 7.6 for SSs and 8.2 compared with 7.8 for SKs, respectively). In Vietnam, FLWs’ motivation scores were similar for both areas.

The number of households visited by FLWs was higher in A&T-I areas than in A&T-NI areas in Bangladesh, and the number of clients who were reached with IYCF services from heath facility was also higher in A&T-I areas in Vietnam (Supplemental Table 4c). The quality of counseling service, reflected by the number of topics delivered during home visits or during the counseling sessions, was greater for A&T-I areas in both areas. Total service delivery scores were significantly greater for A&T-I compared with A&T-NI areas, for SKs (5.4 compared with 3.9) and SSs (6.7 compared with 4.4 scores) in Bangladesh, and for HS (6.8 compared with 4.5) and VHWs (6.2 compared with 3.8) in Vietnam (Figure 2).

#### Performance measured at end-user level

Contact with FLWs was high in Bangladesh (91.3% of women were visited at home by SSs, and 48.9% were visited by SKs), whereas use of the health facility in Vietnam was lower (55% reported visit to A&T franchise facility in intensive areas). Mothers in Bangladesh reported receiving 8.5 home visits by SSs and ∼2.0 visits by PKs, whereas mothers in Vietnam visited the health facilities for counseling an average of 2.3 times (Supplemental Table 5a). Overall utilization scores were higher in A&T-I compared with A&T-NI areas (5.2 compared with 1.5 in Bangladesh and 6.0 compared with 2.4 in Vietnam, respectively) (Figure 2).

In both countries, maternal IYCF-related knowledge was higher in A&T-I compared with A&T-NI areas for the overall knowledge scores (6.4 compared with 5.6 in Bangladesh and 6.0 compared with 5.1 in Vietnam, respectively) (Figure 2) and for several knowledge items (Supplemental Table 5b).

### Influence of individual characteristics on FLW performance

In Bangladesh, most of the FLWs’ and maternal characteristics (except for job duration and SES) were not associated with performance, measured at either the FLW level or the end-user level (**Table 2**). Compared with FLWs with short job duration (<2 y), FLW with 2–5 y of work experience had lower motivation (β = −0.14 out of 10 scores), and those who worked for ≥10 y had higher service delivery scores (β = 1.36). In addition, mothers with higher SES scores had higher knowledge about IYCF.

**TABLE 2:** Associations between individual characteristics and performance^1^

	Bangladesh					Vietnam				
	Performance measured at			Performance measured at		Performance measured at			Performance measured at	
	FLW levels (*n* = 198)			mother's level (*n* = 198)		FLW level (*n* = 60)			mother's level (*n* = 60)	
	Knowledge	Motivation	Service delivery	Utilization	Knowledge	Knowledge	Motivation	Service delivery	Utilization	Knowledge
Frontline workers characteristics										
Age	0.01	0.01	−0.01	−0.01	0.01	−0.02	0.03	−0.07	0.01	−0.01
Education^2^										
No schooling (reference)	—	—	—	—	—	—	—	—	—	—
Primary school	0.13	0.08	0.46	0.11	0.01	—	—	—	—	—
Secondary school	0.19	0.19	0.74	−0.26	0.04	—	—	—	—	—
High school	—	—	—	—	—	—	—	—	—	—
College/university	—	—	—	—	—	0.36	−0.49*	−0.49	−0.01	0.09
Job duration (<2 y is reference), y										
2 to <5	0.14	−0.14*	0.46	0.02	−0.10	0.15	0.33	1.18	0.08	0.21
5 to <10	0.19	0.05	0.46	−0.09	−0.10	0.04	0.12	−0.51	0.17	0.33
10 to <15	0.10	−0.09	1.36**	−0.06	−0.08	−0.09	0.45*	0.68	0.15	0.20
≥15	—	—	—	—	—	0.11	0.34	1.18	0.10	0.30
Maternal characteristics										
Age	—	—	—	0.05	−0.01	—	—	—	1.39***	0.42***
Education	—	—	—	−0.19	−0.03	—	—	—	−0.64***	−0.13
SES	—	—	—	0.15	0.25**	—	—	—	−1.22*	−0.06
Decision-making power	—	—	—	0.08	−0.00	—	—	—	−1.84***	−0.51
Time pressure	—	—	—	−0.06	−0.06	—	—	—	−0.69**	0.03

1 Values are coefficients. FLW, frontline worker; SES, socioeconomic status.

2 The reference for Bangladesh is no schooling; the reference for Vietnam is high school. ^+^, *, **, ***Significant difference between intensive and non-intensive areas, accounting for geographic clustering: *P < 0.05, **P < 0.01, ***P < 0.001.

In Vietnam, FLW motivation was negatively associated with educational level (β = −0.49) but positively associated with longer job duration (β = 0.35–0.45) (Table 2). Mothers with higher education, SES, decision-making power, and time pressures were less likely to use the services, whereas those with older age were more likely to use the IYCF services and had higher IYCF knowledge scores.

### Path analyses on the association between intervention design elements and performance

**FIGURE 3:**
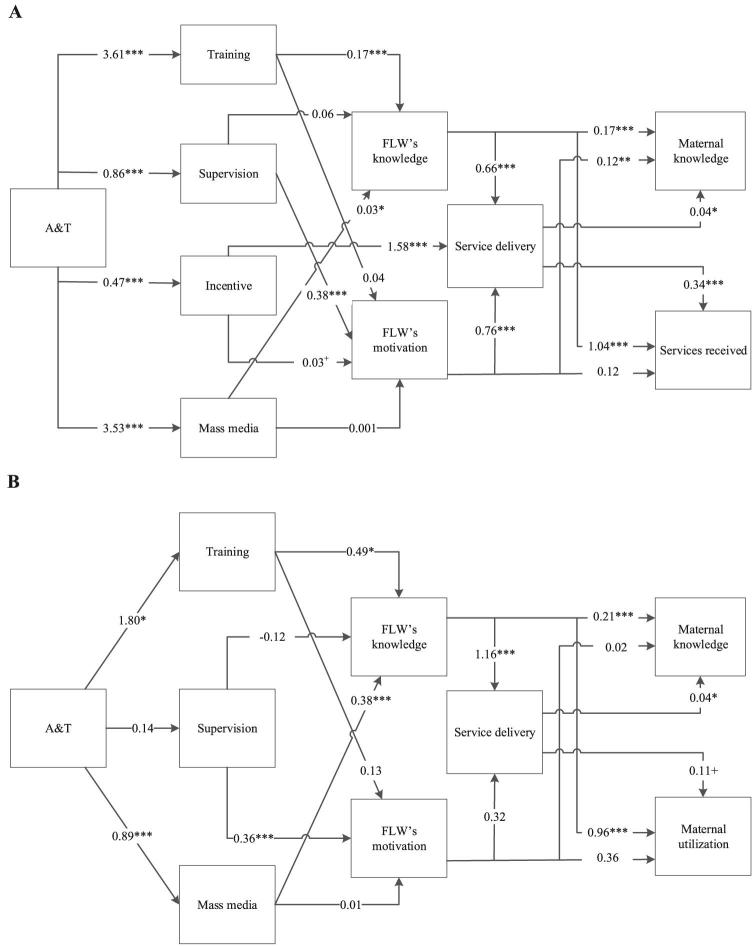
Paths from exposure to performance outcomes measured at the FLW and end-user levels. Bangladesh SS (A) and Vietnam HS (B). Values are coefficients. ^+^, *, **, ***Significant difference between intensive and non-intensive areas, accounting for geographic clustering: ^+^*P *< 0.10, *P < 0.05, **P < 0.01, ***P < 0.001. A&T, Alive & Thrive; FLW, frontline worker; HS, health staff; SS, Shasthya Sebika.

Individual characteristics that were significantly associated with performance were included as covariates in the structural equation analyses to examine the paths between the intervention design elements and performance at the end-user level (maternal knowledge and service utilization) through performance at the FLW level (knowledge, motivation, and service delivery). In Bangladesh, FLWs in the A&T-I areas showed higher training (β = 3.61), supportive supervision (β = 0.86), incentives (β = 0.47), and exposure to mass media (β = 3.53) (**Figure 3**). Whereas training and mass media exposure were positively associated with FLW knowledge (β = 0.17 and 0.03, respectively), supervision was associated with FLW motivation (β = 0.38) and incentives were associated with service delivery (β = 1.58). Both FLW knowledge and motivation were directly associated with higher maternal knowledge (β = 0.17 and 0.12, respectively) and service received (β = 1.04 and 0.12, respectively). They were also indirectly associated with end-user outcomes through higher FLW service delivery. Similar results were observed for Vietnam except that we did not observe the association between FLW motivation and service delivery or service utilization and maternal knowledge.

## Discussion

This article examined the intervention design elements intended to improve performance measured at both service-provider (FLW knowledge, motivation, and service delivery) and end-user levels (service utilization and maternal knowledge) and the paths that link these intervention design elements and performance outcomes. FLWs in A&T-I areas received higher training, supportive supervision, and mass media scores related to IYCF compared with those in A&T-NI areas. These elements, in turn, were associated with higher knowledge and motivation of FLWs, leading to improved service delivery. Furthermore, the differences in performance measured at FLW levels contributed to the improvement of maternal service received/utilization and knowledge on IYCF.

Our results reinforce the importance of training for developing and maintaining health worker competencies to deliver quality services (35). Although the training frequency, intensity, and content differed between Bangladesh and Vietnam, training sessions in both countries offered essential knowledge, counseling technique, and problem-solving skills related to IYCF for FLWs. The higher training exposure and quality in A&T-I areas led to improved performance indirectly through increased knowledge among FLWs. In addition, exposure to mass media was associated with shifting knowledge of FLW, suggesting that combining different intervention design elements leads to greater changes in FLW capacities. The magnitude of influences of different intervention design elements differed by country. In Bangladesh, most of the FLW knowledge change was driven by training rather than mass media exposure (the coefficient from path analyses between training and FLW knowledge was more than 5 times the magnitude of the coefficient for mass media exposure and FLW knowledge), whereas in Vietnam the magnitude of mass media effect was ∼80% of training effects (β = 0.38 compared with 0.49, respectively). Despite the encouraging results of training, however, substantial knowledge gaps on some IYCF topics remained (e.g., the reasons the mother should breastfeed her baby, timely introduction of complementary foods, or feeding during illness), suggesting a need for more tailored and frequent training to equip workers to more effectively and accurately communicate messages to mothers about IYCF practices.

Supervision is another key intervention design element influencing health workers’ ability to carry out their tasks. Supervision has been reported to be important to increase FLW performance in several studies (36, 37), and weak management and supervision of FLWs were reported as negatively influencing scaling up and sustaining of community health programs (38). Details of the supervision structure and its implementation contributing to success, however, were scarce. Our findings suggest that FLWs were satisfied with the supervision overall; they believed that their supervisors were flexible, supportive, and sympathetic to their concerns. In both countries, supervision had indirect effects on performance through enhanced motivation.

In the 2 A&T countries, FLWs are community-level health staff or volunteers who are recruited from and by the community, enhancing their credibility and accountability, enabling integration with the broader health system, and ensuring the acceptability of the program to the communities (38). These FLWs are assigned tasks and responsibilities related to IYCF counseling in addition to their other routine activities. Although overall motivation and perceived value of work were high, some FLWs felt overburdened and reported lower satisfaction with salary. We found significantly positive associations between motivation and service delivery among volunteers in Bangladesh (β = 0.80), but the association was not significant for health staff in Vietnam. This finding for volunteer FLWs is consistent with the literature showing that extended tasks or more responsibility may enhance FLW motivation because they feel more recognition by their community and sense greater value in their position within the health system (39, 40). In contrast, additional work for HS may contribute to burnout or dissatisfaction, and a high workload could result in lower motivation and ultimately lower performance (7). The SSs in Bangladesh received cash performance-based incentives, whereas the VHWs in Vietnam received certificates of recognition as acknowledgment of good work performance, but they showed similar motivation and performance, indicating the important roles of both financial and nonfinancial incentives on FLW motivation and job performance (41, 42). Because these incentives were not randomly assigned and were implemented with different types of FLWs (with different job descriptions and tasks) in different contexts, we were not able to compare the influences of financial and nonfinancial incentives on outcomes.

Measures of performance at the end-user level, which incorporate service exposure and quality, are further influenced by a myriad of factors at both provider and user levels; they are important for verifying and determining whether performance indicators reported by FLWs are indeed associated with mothers’ reports of services received. Given that FLWs provided services through home visits and in communities in Bangladesh, exposure was much higher than in Vietnam, where mothers were required to visit health facilities to receive services. In both countries, however, we observed strong linkages between performance indicators measured at the FLW level and performance measured at the end-user level. FLW knowledge and service delivery were particularly associated with maternal knowledge and service utilization, which confirms our hypotheses that higher FLW knowledge and better service delivery will translate into greater maternal service use and knowledge.

FLWs in A&T-I and A&T-NI areas in each country share similar broad contextual factors (health system and community links, and other resources and logistics), although individual characteristics may be different. Gender of FLWs has been reported to have an influence on performance, including better counseling skills (10) and retention of patients among females (43) but better record-keeping among males (10). In our study, all FLWs in Bangladesh and 80% of FLWs in Vietnam are women, and no differences were observed for knowledge, motivation, or job performance between women and men in Vietnam. Evidence regarding the influence of FLW education level was mixed; some studies have shown that higher education is related to appropriate use of job aids and appropriate counseling, leading to higher performance (10), but higher education may also lead to a higher dropout rate (44). We found that education was not associated with performance in Bangladesh but found a negative association between higher education and motivation among HS in Vietnam. The difference in results regarding education could be due to the difference in education levels: FLWs in Bangladesh had only high school education as the highest level, whereas most FLWs in Vietnam had a college or university education, and the relation may only emerge at this higher cutoff of education. The other FLW characteristic related to performance was longer job duration, which was positively associated with higher service delivery in Bangladesh and higher motivation in Vietnam.

At the end-user level, there was evidence of individual factors that were associated with performance, especially in the Vietnam context. For example, Vietnamese mothers with higher education, higher SES, more decision-making power, and more time pressure had lower utilization of the counseling service in health facilities. This is in contrast with the finding from previous literature that resource constraints negatively affect health service utilization (45–48). Although preventive child health services in Vietnam, including IYCF counseling services, are provided free of charge, more educated and wealthier mothers may seek service at private or higher-level facilities instead of at commune health centers (49). Our findings draw attention to individual factors at both FLW and end-user levels that may limit performance and the need to address these limitations on FLW performance during intervention design and implementation.

Data from different samples of FLWs in 2 countries with the use of similar questionnaires and study methods allowed for a strong comparison across countries. The measures used to capture FLW knowledge, motivation, and performance were validated and locally adapted by taking cultural and social factors into consideration. Although we did not examine cross-country equivalence of measures, we standardized all scales for intervention design elements and performance outcomes to a range from 0 to 10 for comparability. By analyzing differences between the A&T-I and A&T-NI areas within a rigorously designed program evaluation, this study strengthens the plausibility of the observed impacts on FLW performance to deliver nutrition interventions. In addition, the path analyses allowed us to examine the relations from different intervention design elements to performance outcomes measured at both FLW and end-user levels. The measurement at end-user levels provided objective measures of performance and helped mitigate potential bias due to self-report from FLWs. Our study contributes to the growing literature on factors to strengthen the human resources within health systems, particularly for FLWs in LMICs. Consistent with findings from a review of community health worker programs from 19 studies in 16 countries (38), our study calls for effective program design and management (including adequate training, supervision, motivation, and incentives) that are appropriate for community needs and well integrating with the broader environment, for scaling up and sustaining of IYCF programs in LMICs. Lessons learned from this intervention, together with training manuals, job aids, and other implementation manuals, were shared with and adopted by the government in both countries with the intent to integrate and reinforce IYCF interventions delivered through routine health care services (24, 26–28). Program implementation was scaled up to >90% of the subdistricts in Bangladesh, with sustained impacts shown after 1 y of the program (50).

Our study has some limitations. Motivation and supervision are subjectively measured by asking FLWs about their perceptions of job motivation and supportive supervision, which may introduce response bias due to sociocultural norms. Criticism of superiors is highly discouraged in Asian cultures, including in Bangladesh and Vietnam; thus, FLWs may be reluctant to complain about their jobs or supervisors. The lack of direct observations of FLWs’ activities limited our full understanding of FLWs’ performance. Although objective measures of assessment (by observations of activities or shadowing) are thought to be better than subjective ones, they have cost implications and thus could not be applied in our study. Recall bias is possible due to self-reported measures of quantity and quality of service provision by FLWs in Bangladesh, but this issue was less of a concern in Vietnam because FLWs had an opportunity to check on their log books in health facilities during the interview. Finally, recall bias in women with children <2 y may have affected their responses to questions about the number of visits received. For the content of counseling, to minimize timing of recall, we asked women which topics they discussed during their last visit.

## Conclusions

FLWs play a critical role in the delivery of primary health care services in developing countries, particularly in providing preventive services such as health and nutrition counseling. Our findings show that enhancing training, supervision, and mass media exposure can be implemented at large scale and contribute to improved performance in service delivery by improving FLW knowledge and motivation, which in turn positively influence mothers’ service utilization and IYCF knowledge. In balancing priorities and resource constraints, these elements and mechanisms related to service provision should be considered by policymakers and program implementers as essential when developing intervention programs.

## Supplementary Material

nzz070_Supplemental_File

## Notes

This study was funded by the Bill & Melinda Gates Foundation, through Alive & Thrive, managed by FHI 360. Additional financial support to the evaluation study was provided by the CGIAR Research Program on Agriculture for Nutrition and Health (A4NH), led by the International Food Policy Research Institute.

Author disclosures: PHN, SSK, LMT, PM, and EAF, no conflicts of interest.

Supplemental Tables 1–5 are available from the “Supplementary data” link in the online posting of the article and from the same link in the online table of contents at https://academic.oup.com/cdn/.

Abbreviations used: A&T, Alive &Thrive ; HS, Health staff; FLW, frontline health worker; IYCF, Infant and young child feeding; LMICs, low- and middle-income countries; NGO, nongovernmental organization; PK, Pushti Kormi; SES, socioeconomic status; SK, Shasthya Kormi; SS, Shasthya Sebika; VHW, village health worker.
