# Supplementary material for: Intervention Design Elements Are Associated with Frontline Health Workers’ Performance to Deliver Infant and Young Child Nutrition Services in Bangladesh and Vietnam
Source: Curr Dev Nutr. 2019 Jul 10;3(8):nzz070. doi: 10.1093/cdn/nzz070 (PMC6642067; doi:10.1093/cdn/nzz070)
Supplement: nzz070_Supplemental_File [file nzz070_supplemental_file.docx]

**Supplemental Table 1. Description of differences between program groups in Bangladesh**

| **Intervention component** | **A&T Non-intensive** | **A&T Intensive** |
| --- | --- | --- |
| *Training* | Standard training related to maternal and child health programs. Cover some aspects of IYCF. | Similar standard training plus A&T intensive training  ***Full training:***  Training for field staff and frontline workers: 3 days of class training, 1 day of field practice for SS, 3 days of field practice for SK and PK.  The training covers essential information to increase knowledge of IYCF and strengthen skills in counseling, coaching, managing feeding problems, and demonstrating good feeding practices.  Training used mixed interactive methods: Lecture, video on IYCF, hands-on training, role play, practical sessions, one-to-one counseling practice, experience sharing, large and small group discussion, and practice in the community counseling mothers.  ***Monthly Meeting and Refresher Training***  FLWs meet once a month to exchange of lessons learned, planning for the next month, and providing refresher training, as needed, on any health topic. The purpose of the refresher training is to introduce new topics, fill knowledge gaps, and/or solve problems  identified during the monthly observation by supervisors. |
| *Supportive supervision* | Standard supervision by BRAC health care systems | Several levels of supervision are used to guide, support, and motivate staff to perform their assigned tasks   - District managers supervise sub-district managers - Sub-district managers supervise program organizers - Program organizers supervise FLW (1 program organizer supervises 6-8 IYCF promoters). - FLWs (SK & IYCF promoter) supervise other frontline volunteers (1 SK supervises 10-12 SS; 1 IYCF promoter supervises the IYCF-related work of 10-12 SS). |
| *Performance based incentives* | Standard incentive indicators for SS:  - Identification of pregnancy  - Early initiation of breastfeeding | Standard incentives for SS with 4 additional indicators:   - Mother of child <6 months practiced EBF in previous 24 hours - SS demonstrated preparation of complementary foods in 7th month - SS observed and counseled on feeding a sick child - Handwashing station observed near place of child feeding |
| *Interpersonal counseling* | Standard care and nutrition counseling   - SS: in charge of 250-300 households in their community; visit households monthly, provide health products related to maternal and child health, give messages for 10 key health topics including breastfeeding and immunization, refer for child illnesses, help identify pregnancies and link women to ANC, be present at or soon after a delivery. - SK: in charge of 2500-3000 households, provide ANC/PNC to mothers who choose to use BRAC’s services, and motivate mothers to deliver at a health facility. | Standard care (as described in non-intensive), plus added *intensified* counseling:   - SK: provided additional emphasis on early initiation, no pre-lacteals and EBF, animal source foods, feeding quantity during ANC and PNC services - SS: Facilitate initiation of BF within one hour, counseling on IYCF during monthly door-to-door visits to all households with children under two years of age - PK: Counseling on to address difficulties, complete volunteer records of home visits, train mothers at home on complementary feeding at six months, and ensure the following schedule of contacts with mothers during the first two years: monthly from 0 to 8 months, every other month to 12 months, one more visit each between 15-18 and 23-24 months - Additional emphasis given to IYCF at community meetings facilitated by BRAC’s ANC/PNC provider in the community with pregnant and recently delivered women |
| *Community mobilization* | Basic community mobilization through local meetings on topics such as family planning, pregnancy registration, and antenatal care | - Local meetings organized by BRAC managers to introduce the A&T program and activities of workers, targeted to school teachers, adolescents, religious leaders, village doctors, district hospital/clinic doctors, community elites on the topic of nutrition and IYCF; fathers’ meetings added with emphasis on handwashing linked to complementary feeding. - Village theater shows for the general public to generate discussion about the importance of nutrition and IYCF; role of worker |
| *Mass media* | TV and radio Spots: 7 IYCF focused TV commercials aired via four most watched (by rural mothers) national channels and programs 6 to 12 times a day on three days of the week; and radio stories based on TVCs aired on four radio stations and news/sports programs targeted to male audiences | - Same as non-intensive - Added screening of same TV spots plus a cartoon film on IYCF (Meena) via community-based video shows in areas where TV reach was lower (“media dark” strategies) accompanied by community dialogue and quiz shows |
|  | 1. Early Initiation of Breastfeeding: 42’’   <https://www.aliveandthrive.org/resources/tv-spot-early-initiation-of-breastfeeding-bangladesh/>   1. 2. Insufficient Milk: 62’’   <https://www.aliveandthrive.org/resources/tv-spot-perception-of-insufficient-milk-bangladesh/>   1. 3. Father’s Involvement: 43’’   <https://www.aliveandthrive.org/resources/tv-spot-fathers-involvement-bangladesh/>   1. 4. Animal Food: 42’’   <https://www.aliveandthrive.org/resources/tv-spot-animal-food-bangladesh/>   1. 5. Feeding Quantity” 63’’   <https://www.aliveandthrive.org/resources/tv-spot-feeding-quantity-bangladesh/>   1. 6. Poor Appetite: 42’’   <https://www.aliveandthrive.org/resources/tv-spot-poor-appetite-bangladesh/>   1. 7. IYCF plus handwashing: 20’’   <https://www.aliveandthrive.org/resources/tv-spot-iycf-plus-handwashing-bangladesh/> | |

**Supplemental Table 2. Description of differences between program groups in Vietnam**

| **Intervention component** | **A&T Non-intensive** | **A&T Intensive** |
| --- | --- | --- |
| *Training* | Standard training related to maternal and child health programs. Cover some aspects of IYCF. | Similar standard training plus A&T intensive training  ***Full training:***   - Franchise operations: 1-day training delivered to 1,500 franchise managers - IYCF services: 5-day training delivered to 3,300 franchise health staff - IYCF mobilization: 3-day training delivered to 15,112 village health workers   The training covers essential information to increase knowledge of IYCF and strengthen skills in counseling, coaching, managing feeding problems, and demonstrating good feeding practices.  Training used mixed interactive methods: Lecture, video on IYCF, hands-on training, role play, practical sessions, one-to-one counseling practice, experience sharing, large and small group discussion, and practice in the community counseling mothers.  ***Monthly Meeting and Refresher Training***   - Three-day refresher training in 2013 - FLWs meet once a month to exchange of lessons learned, planning for the next month, and providing refresher training. The purpose of the refresher training is to introduce new topics, fill knowledge gaps, and/or solve problems identified during the monthly observation by supervisors. |
| *Supportive supervision* | Standard government supervision system. Do not focus on IYCF | The Vietnam franchise has a three-tiered management structure to to provide guidance, support, and motivate FLWs to perform their assigned tasks   - Franchisor (national level): The A&T project in partnership with National Institute of Nutrition functions as the national franchisor with overall operational oversight and authority - Sub franchisor (provincial and district level) included provincial and district management boards - Franchisee (facility level): directly supervise health staff and village health workers |
| *Incentives* | None | FLWs in Vietnam received certificates of recognition at the end of each year by the program |
| *Interpersonal counseling* | Standard government health services provided at health centers including:   - Provision of at least 3 ANC visits - Weighing children under 5y twice a year - Food demonstrations - Basic nutrition counseling on child feeding - Routine immunization - National vitamin A supplementation and micronutrient days to raise awareness of micronutrients and encourage consumption of micronutrient-rich foods/supplements | Standard government health services (same as non-intensive)   - Added intensive structured package of 8 BF counseling sessions (either group or individual) at social franchise set up at government health centers: - Preparation for EBF through three counseling sessions during the 3rd trimester of pregnancy - Personalized support for initiation of BF at the time of delivery (one contact) - Support and management of EBF through four counseling sessions during first four months of infancy - Use of leaflets, mother-and-child booklets, and distribution of promotional items to mothers who attended counseling sessions (face cloths, raincoats) |
| *Community mobilization* | Village health workers mobilize communities to attend routine health services. | Village health workers visit homes to deliver invitation cards to the counseling sessions |
| *Mass media* | **TV Spots: 4** TV commercials aired via 18 national & provincial channels  **Out of home**   - Sign boards promoting EBF and/or use of the counseling facility in 15 provinces - LCD TV Screens showing the A&T TV spots in supermarkets & hospitals   **Digital**   - Website for the social franchise model (Mat troi be tho) - MTBT fan page - Online digital posters on other popular websites - Mobile phone application | **TV spots**   - Same as non-intensive   **Out of home**   - Same as non-intensive, with added loudspeaker announcements (5 loudspeaker scripts)   **Digital**   - Same as non-intensive   **Others**   - Print materials: posters on breastfeeding at health centers; |
|  | 1. No water: 45’’   <http://aliveandthrive.org/resources/tv-spot-no-water-viet-nam/>   1. Nurse more: 45’’   <http://aliveandthrive.org/resources/tv-spot-breastmilk-only-viet-nam/>   1. Iron rich foods” 30’’   <https://www.aliveandthrive.org/resources/tv-spot-iron-rich-foods-viet-nam/>   1. Promotion of Mat Troi Be Tho franchises: 17’   <http://aliveandthrive.org/resources/tv-spot-promotion-of-mat-troi-be-tho-franchises-viet-nam/> | |

**Supplemental Table 3. Exposure to intervention design elements among frontline health workers, by program group, in Bangladesh and Viet Nam^1^**

| **Sample Characteristics** | **Bangladesh** | | | | **Viet Nam** | | | |
| --- | --- | --- | --- | --- | --- | --- | --- | --- |
|  | **SK** | | **SS** | | **HS** | | **VHW** | |
|  | **A&T-I** | **A&T-NI** | **A&T-I** | **A&T-NI** | **A&T-I** | **A&T-NI** | **A&T-I** | **A&T-NI** |
| **Training exposure** |  |  |  |  |  |  |  |  |
| Main training received |  |  |  |  |  |  |  |  |
| None | 3.92 | 50.00*** | 3.54 | 54.29*** |  |  |  |  |
| Between 1-12 months | 19.61 | 28.33 | 18.18 | 24.76 | 51.67 | 38.33 | 65.97 | 68.89 |
| Between 12-24 months | 15.69 | 11.67 | 23.74 | 8.57** | 5.00 | 8.33 | 16.67 | 2.96*** |
| More than 24 months ago | 60.78 | 10.00*** | 54.55 | 12.38*** | 43.33 | 53.33 | 17.36 | 28.15* |
| Refresh training received between 1-12 months | 100 | 100 | 95.43 | 33.33*** |  |  |  |  |
| Training topics^2^ |  |  |  |  |  |  |  |  |
| Group counseling skills | 26.53 | 20.00 | 29.32 | 29.17 | 88.24 | 75.00 | 83.70 | 75.19 |
| Individual counseling skills |  |  |  |  | 91.18 | 78.57 | 71.85 | 70.54 |
| Breastfeeding positioning and attachment | 42.86 | 10.00** | 51.83 | 14.58*** | 100.0 | 82.14** | 95.56 | 84.50*** |
| Expressing and storing breast milk | -- | -- | 57.98 | 11.43*** | 94.12 | 75.00* | 93.33 | 72.09*** |
| Problem solving for difficulties in IYCF | 38.78 | 6.67** | 34.03 | 25.00 | 88.24 | 67.86 | 80.00 | 61.24** |
| Food demonstrations | -- | -- | -- | -- | 82.35 | 53.57* | 94.07 | 81.40* |
| Feeding techniques | 42.86 | 10.00** | 47.64 | 18.75* | 85.29 | 60.71* | 85.19 | 72.09*** |
| Feeding sick children | 22.45 | 13.33 | 39.27 | 22.92 | 85.29 | 67.86 | 82.96 | 68.99** |
| No of IYCF information received from training | 4.53 ± 2.10 | 1.72 ± 1.40*** | 4.83 ± 1.69 | 1.18 ± 1.18*** | 4.94 ± 4.66 | 3.10 ± 3.73** | 6.71 ± 3.36 | 5.37 ± 3.52*** |
| **Mass media exposure** |  |  |  |  |  |  |  |  |
| Ever seen TV spots | 88.24 | 85.00 | 87.37 | 58.10** | 95.0 | 85.0* | 84.38 | 73.65* |
| No of message recalled from TV spots | 5.96 ± 3.36 | 3.58 ± 2.80*** | 5.42 ± 3.39 | 1.92 ± 2.67*** | 3.24 ± 1.68 | 2.29 ± 1.34** | 2.66 ± 1.37 | 2.37 ± 1.33* |
| **Supervision received** |  |  |  |  |  |  |  |  |
| My supervisor keeps me informed about the follow-up of my concerns/worries | 4.6 ± 0.8 | 4.6 ± 0.8 | 4.6 ± 0.6 | 4.3 ± 0.8* | 4.0 ± 0.8 | 4.0 ± 1.0 | 4.4 ± 0.8 | 4.4 ± 0.8 |
| My supervisor takes my concerns into account when planning activities that involve me | 4.4 ± 1.0 | 4.2 ± 1.0 | 4.5 ± 0.7 | 4.2 ± 0.8* |  |  |  |  |
| When I make a mistake on the job, my supervisor scolds me (reverted) | 4.0 ± 1.4 | 3.6 ± 1.4 | 4.0 ± 1.2 | 3.8 ± 1.3 | 3.2 ± 1.0 | 3.0 ± 1.0 | 3.6 ± 1 | 3.6 ± 1 |
| My supervisor praises me when I do something really well | 4.6 ± 0.8 | 3.6 ± 1.2 | 4.7 ± 0.7 | 3.8 ± 1.1*** | 3.4 ± 1.0 | 3.4 ± 1.0 | 3.6 ± 1 | 3.8 ± 0.8 |
| I feel that my supervisor takes my concerns up to the higher management level | 4.2 ± 1.2 | 3.4 ± 1.2 | 4.1 ± 1.1 | 3.3 ± 1.1** |  |  |  |  |
| My supervisor uses times when I don’t perform well as opportunities to help me improve my skills | 4.6 ± 0.8 | 4.2 ± 1.0 | 4.6 ± 0.7 | 3.8 ± 1.0*** | 3.8 ± 0.8 | 3.6 ± 0.8 | 4.2 ± 0.8 | 4.2 ± 0.8 |
| I feel well informed by my supervisor about changes in the program activities | 4.6 ± 0.4 | 4.4 ± 0.4 | 4.5 ± 0.6 | 4.3 ± 0.5* | 4.0 ± 0.6 | 4.0 ± 1.0 | 4 ± 0.8 | 4.2 ± 0.8 |
| My supervisor ensures that I have enough of the supplies that I need to do my daily work | 4.4 ± 0.8 | 4.2 ± 0.8 | 4.5 ± 0.6 | 4.4 ± 0.5 | 3.8 ± 0.8 | 3.6 ± 0.6 | 4 ± 0.6 | 4 ± 0.6 |
| My supervisor works with me to identify solutions to program activity related problems | 4.6 ± 0.6 | 4.4 ± 0.8 | 4.6 ± 0.6 | 4.3 ± 0.5** | 4.0 ± 0.6 | 4.0 ± 0.8 | 4.2 ± 0.6 | 4.2 ± 0.6 |
| I feel that my supervisor is sympathetic to my problems/cares about my problems | 4.6 ± 0.6 | 4.4 ± 0.8 | 4.6 ± 0.6 | 4.3 ± 0.6* | 4.0 ± 0.6 | 3.8 ± 0.6 | 4.2 ± 0.6 | 4.4 ± 0.6 |
| My supervisor gives me enough guidance and structure to help me do my job | 4.6 ± 0.6 | 4.4 ± 0.6 | 4.6 ± 0.6 | 4.3 ± 0.5** | 3.8 ± 0.6 | 3.8 ± 0.6 | 4.2 ± 0.6 | 4.4 ± 0.6 |
| My supervisor informs me about upcoming trainings/meetings, etc., in a timely fashion |  |  |  |  | 4.0 ± 0.6 | 4.2 ± 0.6 | 4.4 ± 0.6 | 4.4 ± 0.6 |
| My supervisor respects my fixed monthly activities when planning other meetings |  |  |  |  | 4.2 ± 0.6 | 4.2 ± 0.8 | 4.2 ± 0.6 | 4.4 ± 0.6 |
| My supervisor helps me to organize my time and activities in an efficient manner |  |  |  |  | 4.0 ± 0.8 | 3.8 ± 0.8 | 4.2 ± 0.8 | 4.2 ± 0.8 |
| When I disagree with my supervisor I feel safe to express my opinion |  |  |  |  | 3.6 ± 0.8 | 3.4 ± 0.6 | 3.8 ± 1 | 3.8 ± 0.8 |
| My supervisor takes into account/considers my suggestions to improve things |  |  |  |  | 4.0 ± 0.6 | 3.8 ± 0.8 | 4.2 ± 0.6 | 4.2 ± 0.6 |
| ***Supervision score (range 0 – 10)*** | ***8.98 ± 0.92*** | ***8.27 ± 1.01***** | ***9.02 ± 0.86*** | ***8.20 ± 0.79****** | ***7.72 ± 0.86*** | ***7.55 ± 0.96*** | ***8.21 ± 0.80*** | ***8.31 ± 0.72*** |
| **Incentive** |  |  |  |  |  |  |  |  |
| Ever received incentive |  |  | 99.0 | 55.2*** |  |  |  |  |
| Received monthly incentive |  |  | 95.45 | 50.48*** |  |  |  |  |
| *Activities that FLWs receive incentive for* |  |  |  |  |  |  |  |  |
| No of mothers practice early initiation of BF |  |  | 94.4 | 52.4*** |  |  |  |  |
| No of mothers practice EBF |  |  | 80.3 | 21.9*** |  |  |  |  |
| No of food demonstration sections |  |  | 80.8 | 3.8*** |  |  |  |  |
| No of mothers fed the recommended frequency of family cooked food according to the child age |  |  | 17.7 | 1.0*** |  |  |  |  |
| No of mothers fed the correct quantity of family cooked food according to the child age |  |  | 13.1 | 0.0*** |  |  |  |  |
| No of mothers fed animal source food to children |  |  | 4.0 | 0.0** |  |  |  |  |
| No of mothers practiced hand washing with soap |  |  | 69.7 | 0.0*** |  |  |  |  |

^1^Values are means ± SDs or percentages, ^2^Among FHWs who received training, *,**,*** Significant difference between intensive and non-intensive areas, accounting for geographic clustering: * *P* < 0.05, ** *P* <0.01, *** *P* < 0.001. A&T-I, Alive & Thrive-Intensive; A&T-I, Alive & Thrive-Non Intensive; CHC, Community Health Center; HS, Health Staff; IYCF, Infant and Young Child Feeding; SK, Shasthya Kormi; SS, Shasthya Sebika; TV, Television; VHW, Village Health Worker.

**Supplemental Table 4A. Knowledge about infant and young child feeding of frontline health workers, by program group, in Bangladesh and Viet Nam^1^**

| **Sample Characteristics** | **Bangladesh** | | | | **Viet Nam** | | | |
| --- | --- | --- | --- | --- | --- | --- | --- | --- |
|  | **SK** | | **SS** | | **HS** | | **VHW** | |
|  | **A&T-I** | **A&T-NI** | **A&T-I** | **A&T-NI** | **A&T-I** | **A&T-NI** | **A&T-I** | **A&T-NI** |
| **Breastfeeding** |  |  |  |  |  |  |  |  |
| Baby should be breastfed during first hours after birth | 98 | 95 | 99.5 | 97.1** | 98.3 | 96.7 | 94.4 | 91.0 |
| Baby should be fed colostrum | 96.1 | 98.3 | 95.5 | 98.1 | 100 | 96.7 | 99.4 | 95.8* |
| Reasons why a baby should be breastfed | 34.5 | 31.4 | 33.5 | 27.3** | 45.4 | 32.6*** | 34.0 | 31.1* |
| Breastfeed baby whenever baby wants | 43.1 | 40 | 47.5 | 45.7 | 95 | 81.7* | 83.1 | 63.5* |
| Even women with small breast can produce milk | 80.4 | 80 | 89.9 | 71.4** | 100 | 90* | 92.5 | 83.8** |
| Mother can produce enough milk even when she is not fed well | 25.5 | 5.0** | 23.2 | 11.4* | 53.3 | 28.3** | 21.9 | 16.8 |
| Frequent BF during the day and night increase milk production |  |  |  |  | 100 | 100 | 95.0 | 96.4 |
| The most common reason to have over-full, hard, sore breasts | 96.1 | 83.3* | 92.4 | 72.4** |  |  |  |  |
| Breastfed more often if mother thinks her baby is not getting enough breast milk | 72.5 | 36.7** | 76.8 | 20.0*** | 75.0 | 53.3* | 46.9 | 28.1** |
| Baby <6 months should not be given water in hot weather | 98 | 91.7* | 97.0 | 63.8*** | 100 | 73.3*** | 91.9 | 72.5*** |
| Mother should continue breastfeeding if she is pregnant | 82.4 | 81.7 | 85.4 | 82.9 | 85 | 76.7 | 70.6 | 67.1 |
| Mother should continue breastfeeding even if she is sick | 86.3 | 85.0 | 85.9 | 79.0 | 98.3 | 75.0*** | 83.1 | 50.9*** |
| Mother should leave breastmilk for baby when she is away | 96.1 | 55.0*** | 94.4 | 24.8*** | 100 | 93.3 | 97.5 | 95.2 |
| Breastfed baby until age of 24 months or beyond | 96.1 | 96.7 | 97.5 | 93.3 | 100 | 93.3* | 93.8 | 83.8** |
| **Complementary feeding** |  |  |  |  |  |  |  |  |
| Timely introduction of complementary food | 27.5 | 53.3** | 35.4 | 65.7*** | 78.3 | 63.3* | 52.5 | 43.7 |
| Age-appropriate feeding frequency | 97.4 | 95 | 97.1 | 96.8 | 91.7 | 83.3 | 87.5 | 85.0 |
| Age-appropriate food quantity for each meal | 96.1 | 93.3 | 98.0 | 93.3 | 93.3 | 81.7** | 84 | 84.4 |
| Dietary diversity (≥4 food groups) |  |  |  |  | 99.4 | 96.7 | 98.8 | 97.8 |
| Know at least one thing mother can do to encourage infant to eat | 96.1 | 98.3 | 99.5 | 100 | 83.3 | 78.3 | 90 | 87.4 |
| Know at least one consequence of not getting enough iron | 92.2 | 95 | 92.4 | 87.6 | 93.3 | 96.7 | 81.3 | 88.0* |
| Vitamin C helps to absorb iron better |  |  |  |  | 76.7 | 40.0*** | 15.6 | 15.0 |
| Foods that rich in Vitamin A |  |  |  |  | 35.3 | 24.2*** | 27.6 | 24.5** |
| Feeding when the child has diarrhea | 39.5 | 37.2 | 39.1 | 36.7 | 44.3 | 36.7 | 38.8 | 38.7 |
| Feeding the child during recovery from illness | 37.9 | 32.5** | 37.0 | 28.4*** | 31.0 | 24.5* | 31.5 | 27.2* |
| ***Knowledge score (range 0 – 10)*** | ***7.46***  ***± 1.18*** | ***6.92***  ***± 1.19**** | ***7.58***  ***± 0.82*** | ***6.48***  ***± 0.88****** | ***8.16***  ***± 0.69*** | ***7.02***  ***± 1.05****** | ***7.01***  ***± 1.03*** | ***6.38***  ***± 1.06****** |

^1^Values are means ± SDs or percentages. *,**,*** Significant difference between intensive and non-intensive areas, accounting for geographic clustering: * *P* < 0.05, ** *P* <0.01, *** *P* < 0.001. A&T-I, Alive & Thrive-Intensive; A&T-I, Alive & Thrive-Non Intensive; HS, Health Staff; IYCF, SK, Shasthya Kormi; SS, Shasthya Sebika; VHW, Village Health Worker.

**Supplemental Table 4B. Motivation of frontline health workers, by program group, in Bangladesh and Viet Nam^1^**

|  | **Bangladesh** | | | | **Vietnam** | | | |
| --- | --- | --- | --- | --- | --- | --- | --- | --- |
|  | **SK** | | **SS** | | **HS** | | **VHW** | |
|  | **A&T-I** | **A&T-NI** | **A&T-I** | **A&T-NI** | **A&T-I** | **A&T-NI** | **A&T-I** | **A&T-NI** |
| **Motivation** |  |  |  |  |  |  |  |  |
| Confident and enjoyment | 4.54 | 4.48 | 4.58 | 4.32** | 4.25 | 4.20 | 4.26 | 4.34 |
| Personal recognition or appreciation | 4.73 | 4.53* | 4.65 | 4.30** | 4.21 | 4.09 | 4.26 | 4.27 |
| Satisfaction with training | 4.08 | 3.92 | 4.13 | 4.03 | 3.88 | 3.84 | 4.05 | 4.00 |
| Satisfaction with salary | 3.28 | 2.73* | 3.78 | 3.22** | 3.00 | 2.98 | 2.67 | 2.84 |
| Support | 4.69 | 4.51* | 4.62 | 4.39*** | 4.47 | 4.46 | 4.47 | 4.37* |
| Work pressure | 3.43 | 3.60 | 3.41 | 3.44 | 3.05 | 3.02 | 2.72 | 2.66 |
| Thought of leaving/ negative attitude | 1.96 | 2.24* | 2.41 | 2.79** | 1.3 | 1.2 | 1.49 | 1.23** |
| ***Total motivation score (0-10)*** | **8.15** | **7.79**** | **8.09** | **7.59***** | **7.65** | **7.60** | **7.76** | **7.86** |

^1^Values are means ± SDs. *,**,*** Significant difference between intensive and non-intensive areas, accounting for geographic clustering: * *P* < 0.05, ** *P* <0.01, *** *P* < 0.001. A&T-I, Alive & Thrive-Intensive; A&T-I, Alive & Thrive-Non Intensive; HS, Health Staff; SK, Shasthya Kormi; SS, Shasthya Sebika; VHW, Village Health Worker.

**Supplemental Table 4C. Frontline health workers’ service delivery related to infant and young child feeding, by program group, in Bangladesh and Viet Nam^1^**

|  | **Bangladesh** | | | | **Viet Nam** | | | |
| --- | --- | --- | --- | --- | --- | --- | --- | --- |
|  | **SK** | | **SS** | | **HS** | | **VHW** | |
|  | **A&T-I** | **A&T-NI** | **A&T-I** | **A&T-NI** | **A&T-I** | **A&T-NI** | **A&T-I** | **A&T-NI** |
| **Performance** |  |  |  |  |  |  |  |  |
| ***Service provision (in a month)*** |  |  |  |  |  |  |  |  |
| No of households visited | 425 ± 129 | 350 ± 143* | 191 ± 120 | 111 ± 105*** |  |  | 18.58 ± 17.57 | 20.53 ± 20.64 |
| No of clients received BF counselling |  |  |  |  | 41.23 ± 47.37 | 13.26 ± 18.18*** |  |  |
| No of clients received CF counselling |  |  |  |  | 31.17 ± 40.06 | 11.18 ± 10.23** |  |  |
| No of health and nutrition forums for mothers and children |  |  |  |  |  |  | 1.80 ± 2.12 | 1.15 ± 0.84 |
| No of food demonstrations conducted |  |  | 5.62 ± 4.97 | 3.65 ± 5.48 | 11.15 ± 10.27 | 3.73 ± 2.33** | 0.43 ± 0.66 | 0.39 ± 0.49 |
| ***Quality of counselling services*** |  |  |  |  |  |  |  |  |
| No of topics delivered in home visits/ counseling during pregnancy |  |  | 2.20 ± 0.72 | 1.82 ± 0.76* | 1.15 ± 0.76 | 0.53 ± 0.70*** |  |  |
| No of topics delivered in home visits/ counseling when child aged 0-5 months |  |  | 3.11 ± 0.91 | 2.31 ± 0.95*** | 1.53 ± 1.11 | 0.68 ± 0.70*** |  |  |
| No of topics delivered in home visits/ counseling when child aged 6-23 months |  |  | 14.37 ± 4.10 | 10.31 ± 4.27*** | 1.98 ± 1.26 | 1.13 ± 1.28*** |  |  |
| Provide advice on maternal nutrition |  |  |  |  |  |  | 81.88 | 80.84 |
| Provide advice on breastfeeding | 70.59 | 46.67* |  |  |  |  | 89.38 | 89.82 |
| Provide advice on CF | 29.41 | 15.0 |  |  |  |  | 86.88 | 79.64* |
| ***Total performance score (0-10)*** | ***5.35 ± 1.78*** | ***3.91 ± 3.91***** | ***6.72 ± 1.90*** | ***4.35 ±1.91****** | ***6.78 ± 2.56*** | ***4.47 ± 2.31****** | ***6.17 ± 2.59*** | ***3.78 ± 1.43****** |

^1^Values are means ± SDs or percentages. *,**,*** Significant difference between intensive and non-intensive areas, accounting for geographic clustering: * *P* < 0.05, ** *P* <0.01, *** *P* < 0.001. A&T-I, Alive & Thrive-Intensive; A&T-I, Alive & Thrive-Non Intensive; BF, Breastfeeding; CF, Complementary feeding; HS, Health Staff; SK, Shasthya Kormi; SS, Shasthya Sebika; VHW, Village Health Worker

**Supplemental Table 5A. Service utilization by mothers, by program group, in Bangladesh and Viet Nam^1^**

| **Sample Characteristics** | **Bangladesh** | | **Viet Nam** | | |  |
| --- | --- | --- | --- | --- | --- | --- |
|  | **A&T-I** | **A&T-NI** | | **A&T-I** | **A&T-NI** | |
| **Quantity of service received** |  |  | |  |  | |
| **SS visits at home** |  |  | |  |  | |
| Ever visited at home | 91.31 | 26.40*** | |  |  | |
| No of times visited during the third trimester of pregnancy | 1.22 ± 1.08 | 0.32 ± 0.77*** | |  |  | |
| No of times visited from delivery to 24 months after birth | 7.40 ± 6.92 | 0.83 ± 2.73*** | |  |  | |
| No of times visited from pregnancy to 24 months after birth | 8.45 ± 6.89 | 1.13 ± 2.96 | |  |  | |
| No of times visited in the last 6 months | 4.31 ± 2.85 | 0.56 ± 1.54*** | |  |  | |
| No of times visited in the last 30 days | 1.12 ± 0.80 | 0.14 ± 0.47*** | |  |  | |
| **SK visits at home** |  |  | |  |  | |
| Ever visited at home by SK | 48.85 | 36.60 | |  |  | |
| No of times visited during the third trimester of pregnancy | 0.64 ± 0.85 | 0.47 ± 0.83* | |  |  | |
| No of times visited from delivery to 24 months after birth | 1.25 ± 2.37 | 0.74 ± 1.64* | |  |  | |
| No of times visited from pregnancy to 24 months after birth | 1.96 ± 2.96 | 1.22 ± 2.25* | |  |  | |
| No of times visited in the last 6 months | 0.97 ± 1.97 | 0.58 ± 1.29* | |  |  | |
| No of times visited in the last 30 days | 0.18 ± 0.43 | 0.12 ± 0.37 | |  |  | |
| **Ever attended health forums** | 6.49 | 1.10 | |  |  | |
| **MTBT visits** |  |  | |  |  | |
| Ever visited MTBT counselling room |  |  | | 54.86 | 1.49*** | |
| No of times visited MTBT during pregnancy |  |  | | 1.00 ± 1.60 | 0.02 ± 0.21*** | |
| No of times visited MTBT when child was 0-5 months |  |  | | 0.69 ± 1.19 | 0.01 ± 0.09*** | |
| No of times visited MTBT when child was 6-11 months |  |  | | 0.68 ± 1.13 | 0.01 ± 0.08*** | |
| No of times visited MTBT when child was 12-24 months |  |  | | 0.06 ± 1.39 | 0.01 ±0.08*** | |
| Number of times visited MTBT counseling room from pregnancy to 24 months after birth |  |  | | 2.26 ± 3.40 | 0.03 ± 0.33*** | |
| No of times visited MTBT in the last 6 months |  |  | | 0.83 ± 1.30 | 0.01 ± 0.07*** | |
| **Quality of services received** |  |  | |  |  | |
| Number of messages on IYCF received in the last visit of SS | 1.61 ± 1.14 | 0.14 ± 0.52*** | |  |  | |
| Number of messages on IYCF received in the last visit of SK | 0.56 ± 1.04 | 0.23 ± 0.68*** | |  |  | |
| Number of messages on IYCF received in the last health forum | 0.03 ± 0.20 | 0.01 ± 0.08* | |  |  | |
| Number of messages on IYCF received in the last visit |  |  | | 1.65 *± 1.80* | 0.20 *± 0.73**** | |
| ***Utilization score (range 0 – 10)*** | ***5.19 ± 2.41*** | ***1.46 ± 1.18****** | | ***5.98 ± 3.49*** | ***2.35 ± 1.19****** | |

^1^Values are means ± SDs or percentages. *,**,*** Significant difference between intensive and non-intensive areas, accounting for geographic clustering: + *P*<0.01, * *P* < 0.05, ** *P* <0.01, *** *P* < 0.001. A&T-I, Alive & Thrive-Intensive; A&T-I, Alive & Thrive-Non Intensive; IYCF, Infant and Young Child Feeding; HS, Health Staff; MTBT, Mat troi Be Tho; SK, Shasthya Kormi; SS, Shasthya Sebika; VHW, Village Health Worker.

**Supplemental Table 5B. Knowledge about infant and young child feeding of mothers, by program group, in Bangladesh and Viet Nam^1^**

| **Sample Characteristics** | **Bangladesh** | | **Viet Nam** | | | |
| --- | --- | --- | --- | --- | --- | --- |
|  | **A&T-I** | **A&T-NI** | | **A&T-I** | **A&T-NI** |  |
| **Breastfeeding** |  |  | |  |  |  |
| Baby should be breastfed during first hours after birth | 95.6 | 93.8 | | 87.0 | 78.4*** |  |
| Baby should be fed colostrum | 90.9 | 89.3 | | 97.4 | 92.9*** |  |
| Reasons why a baby should be breastfed | 25.5 | 23.8* | | 23.0 | 20.1*** |  |
| Breastfeed baby whenever baby wants | 40.7 | 36.8 | | 48.4 | 36.3*** |  |
| Even women with small breast can produce milk |  |  | | 89.1 | 83.1** |  |
| Mother can produce enough milk even when she is not fed well |  |  | | 17.7 | 10.6 |  |
| The most common reason to have over-full, hard, sore breasts | 62.6 | 51.4* | |  |  |  |
| Breastfed more often if mother thinks her baby is not getting enough breast milk | 29.3 | 18.3** | | 18.6 | 5.9*** |  |
| Baby <6 months should not be given water in hot weather | 76.1 | 36.4*** | | 65.6 | 29.8*** |  |
| Mother should continue breastfeeding if she is pregnant | 89.0 | 73.0*** | | 44.4 | 33.2** |  |
| Mother should continue breastfeeding even if she is sick | 94.2 | 85.1*** | | 92.6 | 81.1*** |  |
| Mother should leave breastmilk for baby when she is away | 42.2 | 8.9*** | | 52.8 | 21.1*** |  |
| Breastfed baby until age of 24 months or beyond | 91.6 | 85.2* | | 79.8 | 60.5*** |  |
| **Complementary feeding** |  |  | |  |  |  |
| Timely introduction of complementary food | 55.1 | 59.0 | | 49.5 | 43.0** |  |
| Age-appropriate feeding frequency | 92.4 | 91.6 | | 91.5 | 90.1 |  |
| Age-appropriate food quantity for each meal | 96.9 | 90.9** | |  |  |  |
| Dietary diversity (≥4 food groups) |  |  | | 67.6 | 65.3 |  |
| Know at least one thing mother can do to encourage infant to eat | 97.6 | 96.9 | | 91.7 | 88.1* |  |
| Know at least one consequence of not getting enough iron | 28.5 | 22.4** | | 93.2 | 92.3 |  |
| Vitamin C helps to absorb iron better |  |  | | 14.4 | 13.9 |  |
| Foods that rich in Vitamin A |  |  | | 22.5 | 20.9* |  |
| Feeding when the child has diarrhea | 32.5 | 31.5 | |  |  |  |
| Feeding the child during recovery from illness | 19.1 | 18.7 | |  |  |  |
| ***Knowledge score (range 0 – 10)*** | ***6.44***  ***± 0.93*** | ***5.63***  ***± 0.93****** | | ***6.03***  ***± 1.35*** | ***5.09***  ***± 1.18****** |  |

^1^Values are means ± SDs or percentages. *,**,*** Significant difference between intensive and non-intensive areas, accounting for geographic clustering: * *P* < 0.05, ** *P* <0.01, *** *P* < 0.001. A&T-I, Alive & Thrive-Intensive; A&T-I, Alive & Thrive-Non Intensive.
